# Two-injection start regimen of long-acting aripiprazole for bipolar disorder: tips and tricks for better efficacy and tolerability

**DOI:** 10.3389/fpsyt.2025.1627052

**Published:** 2026-01-23

**Authors:** Gulcin Elboga, Irem Surme, Muhammet Sancaktar, Bahadır Demir, Sengul Kocamer Sahin, Abdurrahman Altindag

**Affiliations:** 1 Department of Psychiatry, Gaziantep University Faculty of Medicine, Gaziantep, Türkiye; 2 Department of Psychiatry, Kayseri State Hospital, Kayseri, Türkiye; 3 Department of Psychiatry, University of Health Sciences Training and Research Hospital, Adana, Türkiye

**Keywords:** aripiprazole, bipolar disorder, effectiveness, long-acting injectable, one-injection start, safety, two-injection start

## Abstract

**Objective:**

To comparatively evaluate the efficacy and safety of the two aripiprazole long-acting injectable (LAI) initiation regimens, including one-injection start (OIS) regimen and two-injection start (TIS) regimen, in patients with bipolar disorder (BD).

**Methods:**

A total of 46 BD patients experiencing acute manic episodes with psychotic or mixed features were included in this observational study. Patients were divided into two groups based on the aripiprazole LAI initiation regimen including OIS group (n=23; patients received a single 400 mg LAI injection on Day 1, followed by oral aripiprazole (15–20 mg/day) for 14 days) and TIS group (n=23; patients received two 400 mg doses of aripiprazole LAI administered on the same day together with a single 20 mg oral dose of aripiprazole). Data on patient demographics, clinical characteristics and previous hospitalization were retrieved from hospital records. The effectiveness of the treatment was evaluated based on the Young Mania Rating Scale (YMRS) and Hamilton Depression Scale (HAM-D) scores recorded on Day 1 and Day 15 of the injection. The safety and tolerability of the OIS and TIS regimens were assessed via Udvalg for Kliniske Undersøgelser Side Effect Rating Scale (UKU-SERS) scores recorded on Day 15 of the injection.

**Results:**

At baseline, the rate of previous hospitalization was higher in the OIS group than in the TIS group (60.9% vs. 26.1%, p=0.037). YMRS scores on Day 15 (11.0 ± 5.7 vs. 16.6 ± 6.2, p=0.003) and UKU-SERS Psychiatric adverse effects score on Day 15 (4.4 ± 1.8 vs. 7.5 ± 3.2, p<0.001) were significantly lower in the TIS group than in the OIS group. YMRS scores and HAM-D scores were significantly decreased from Day 1 to Day 15 of injection in both TIS (p= 0.002 and p=0.025, respectively) and OIS (p= 0.002 and p=0.025, respectively) groups, while the change was significantly higher in the TIS group. Linear regression analysis revealed that OIS regimen was a significant risk factor for increase in YMRS Day 15 scores (B: 0.434, p=0.003) and increase in UKU-SERS psychiatric scores (B: 0.526, p<0.001). Logistic regression analysis revealed that OIS regimen was an independent determinant of previous hospitalization history (OR: 4.407, p=0.020), increasing the likelihood of previous hospitalization by 4.407 times compared to TIS regimen. Both regimens had tolerable side effects, with no life-threatening issues.

**Conclusions:**

This study highlights the efficacy and reliability of the TIS regimen in treating BD episodes, potentially facilitating faster clinical recovery by reducing the need for prolonged oral supplementation.

## Introduction

Bipolar disorder (BD) is a chronic mental illness characterized by intense mood shifts with alternating episodes of mania (or hypomania) and depression, leading to significant impairment in vocational, social and occupational functioning of affected individuals (1, 2).

More than 1% of the world’s population is at risk of developing BD throughout their lifetime, while the disorder typically begins in young adulthood with significant impacts on workforce and societal costs (2).

Minimizing problems that may arise during mood episodes, enhancing the functionality of affected individuals, and reducing the risk of relapse are crucial in the optimal management of BD (3). However, poor medication adherence, as one of the major causes of relapses and functional impairment, is observed in up to 64% of BD patients (4). Particularly during acute manic episodes, loss of insight and treatment refusal behaviors severely hinder the continuity of oral antipsychotic use (5, 6).

Long-acting injectable (LAI) antipsychotics have become an important treatment option in this regard, given their favorable tolerability profile and their potential to facilitate treatment continuity, thereby contributing to the prevention of manic relapses and related hospitalizations among patients with bipolar disorder (7–9).

Current guidelines for the treatment of BD indicate aripiprazole amongst the first-line treatment options for acute mania, mixed episodes, and maintenance therapy (10, 11). Additionally, it is considered a first-line treatment option for agitation (10, 11).

Indeed, aripiprazole LAI stands out as an important therapeutic alternative in BD, given its advantageous side-effect profile including fewer metabolic side effects than other second-generation LAI antipsychotics and no prolactin-related side effects (12). Although there are regional differences in the formulations of long-acting injectable (LAI) aripiprazole, the standard one-injection start (OIS) regimen generally consists of administering 400 mg of aripiprazole LAI monthly, along with 10–20 mg of oral aripiprazole daily for 14 days to achieve therapeutic plasma levels (13, 14). However, the requirement to continue oral treatment for 14 days presents a significant challenge, particularly in patient populations with poor medication adherence, and increases the risk of early relapse. In contrast, the two-injection start (TIS) regimen consists of two LAI aripiprazole injections administered on the same day, together with a single oral dose of aripiprazole, thereby eliminating the need for prolonged oral supplementation.

Recently, a simplified aripiprazole initiation regimen was approved by the European Medicines Agency (EMA), namely the two-injection start (TIS) regimen, which involves two aripiprazole 400 mg injections to different muscles plus a single dose of oral aripiprazole 20 mg, all on the same day (14, 15). In a study by Wang et al, OIS and TIS regimens were reported to have similar pharmacokinetic profiles (16). Martiadis et al. noted that initiation strategies for long-acting injectable (LAI) aripiprazole may critically influence clinical outcomes by shaping early plasma exposure and treatment engagement. They emphasized the growing need to compare the TIS and OIS approaches with regard to the speed of onset, the requirement for oral supplementation, and early tolerability. Their study underscored the importance of assessing real-world feasibility and patient-centered outcomes, particularly in manic presentations where adherence tends to be fragile (17). However, the pharmacokinetic differences between the two strategies—particularly in terms of the speed of treatment initiation, early plasma levels, and the need for oral supplementation—hold distinct importance for clinical outcomes. The availability and initiation strategies of long-acting injectable (LAI) aripiprazole formulations vary across regions. This study focuses on aripiprazole once-monthly (aripiprazole maintena), which is widely used in Europe and requires either oral supplementation or a two-injection start regimen during initiation. These regional differences should be considered when interpreting the findings.

The newly approved TIS regimen eliminates the need for 14 days of oral overlap and may therefore provide a faster onset of action by eliminating the need for prolonged oral overlap. While the efficacy and tolerability of the TIS regimen have been studied in patients with schizophrenia, TIS regimen has not been systematically evaluated in the setting of BD along with limited data on the real-world use of the TIS regimen in BD patients (14).

Therefore, this study aimed to compare the efficacy and tolerability of two aripiprazole LAI initiation regimens and to provide clinically relevant insights regarding their real-world implementation during acute treatment.

## Methods

This study was designed as a matched retrospective observational analysis comparing the efficacy and safety of two aripiprazole long-acting injectable (LAI) initiation regimens—the two-injection start (TIS) and the one-injection start (OIS)—in patients with bipolar disorder (BD) experiencing acute manic episodes with psychotic or mixed features. Patient selection was based on a retrospective review of electronic medical records and e-prescription databases of individuals treated at the Department of Psychiatry, Gaziantep University Faculty of Medicine. Eligible patients were identified through digital pharmacy logs and verified clinical documentation confirming aripiprazole LAI initiation. A total of 46 patients (25 males, 21 females) were included in the analysis. All patient identifiers were anonymized prior to data extraction and statistical processing. Patients were divided into two groups based on aripiprazole LAI initiation regimen including OIS group (patients received a single 400 mg LAI injection on Day 1, followed by oral aripiprazole (15–20 mg/day) for 14 days; n=23) and TIS group (patients received two 400 mg doses of aripiprazole LAI administered on the same day together with a single 20 mg oral dose of aripiprazole; n=23). Age between 18 to 65 years, diagnosis of BD with manic episodes according to DSM-5 diagnostic criteria and receiving OIS or TIS initiation regimen of LAI aripiprazole were the inclusion criteria of the study. A primary diagnosis of schizophrenia spectrum or schizoaffective disorder or evidence of severe cognitive impairment, known hypersensitivity to any excipient of aripiprazole or LAI formulation, use of another long-acting injectable antipsychotic within the past 3 months, pregnancy or breastfeeding at the time of data collection were exclusion criteria for the study.

The study was conducted in accordance with the ethical principles stated in the “Declaration of Helsinki” and approved by the Gaziantep University Faculty of Medicine Ethics Committee (Protocol No: 15.11.2023-2023/375). Informed consent was obtained from each patient.

### Group definitions

**Figure 1:**
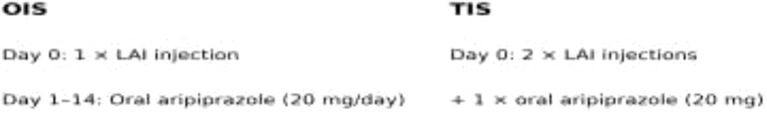
Schematic illustration of the two aripiprazole long-acting injectable (LAI) initiation strategies used in this study. In the one-injection start (OIS) regimen, patients receive a single 400 mg intramuscular injection of aripiprazole LAI on Day 1, followed by oral aripiprazole supplementation (15–20 mg/day) for 14 consecutive days. In contrast, the two-injection start (TIS) regimen involves the administration of two separate 400 mg intramuscular injections of aripiprazole LAI to different muscle sites on the same day, together with a single 20 mg oral dose of aripiprazole.

Patients were divided into two groups according to the aripiprazole LAI initiation regimen documented in their medical records: OIS group (n = 23) received a single 400 mg intramuscular injection of aripiprazole LAI, followed by oral aripiprazole supplementation (15–20 mg/day) for 14 consecutive days to maintain therapeutic plasma levels. TIS group (n = 23) received two separate 400 mg injections of aripiprazole LAI administered to different muscle sites (typically bilateral gluteal muscles) on the same day, along with a single 20 mg oral aripiprazole dose(**Figure 1**). This protocol is consistent with the European Medicines Agency (EMA)–approved accelerated initiation strategy.

Matching was performed based on demographic and clinical characteristics (age, sex, diagnosis subtype, and prior hospitalization) to ensure balanced comparison between groups. Although this was a non-randomized design, the use of matched retrospective sampling allowed for control of confounding variables and reduced potential bias related to treatment selection.

**Figure 1**. Timeline of aripiprazole LAI initiation regimens (OIS vs TIS).

### Clinical setting

The study was conducted at the Department of Psychiatry of Gaziantep University Faculty of Medicine, a tertiary referral center providing both inpatient and outpatient psychiatric services. The clinic serves a large and heterogeneous population, including patients referred from surrounding regions, and routinely manages acute manic and mixed episodes requiring rapid pharmacological stabilization. Long-acting injectable antipsychotics are commonly initiated in both inpatient wards and closely monitored outpatient settings, allowing for real-world implementation of different initiation strategies. This setting reflects routine clinical practice in a university-affiliated public hospital, thereby enhancing the external validity and generalizability of the findings.

### Assessments

Data on patient demographics (age, gender), the diagnosis (psychotic mania or mixed mania), previous hospitalization and the first attack status were retrieved from hospital records. Young Mania Rating Scale (YMRS), Hamilton Depression Scale (HAM-D) and Udvalg for Kliniske Undersøgelser Side Effect Rating Scale (UKU-SERS) were completed by the investigators through documentary review. The effectiveness of the treatment was evaluated based on the YMRS and HAM-D scores recorded on Day 1 and Day 15 of the aripiprazole LAI. The safety and tolerability of the OIS and TIS regimens were assessed via UKU-SERS scores recorded on day 15 after the aripiprazole LAI administration.

Medication adherence, particularly adherence to the 14-day oral aripiprazole supplementation in the OIS group, was not objectively measured in this study.

### Young Mania Rating Scale

The Young Mania Rating Scale (YMRS) is an 11-item clinician-rated scale used to assess the severity of manic symptoms, with total scores ranging from 0 to 60. Higher scores indicate greater manic symptom severity, and scores above 20 are generally considered indicative of moderate to severe mania. The Turkish validity and reliability of the scale have been established previously (17, 18).

### Hamilton Depression Scale

The Hamilton Depression Rating Scale (HAM-D-17) is a clinician-rated scale used to evaluate depressive symptom severity, with total scores ranging from 0 to 53. Higher scores reflect greater depressive symptom severity. The Turkish validity and reliability of the scale have been established (19, 20).

### Udvalg for Kliniske Undersøgelser Side Effect Rating Scale

The UKU-SERS is a semi-structured instrument used to assess adverse effects of psychotropic medications. It consists of psychiatric (psychic), neurologic, autonomic, and other side-effect domains, with items rated from 0 (absent) to 3 (severe). The term “psychic side effects” refers to psychiatric symptom-related adverse effects rather than psychotic symptoms. The Turkish validity and reliability of the scale have been established (21, 22).

### Statistical analysis

Statistical analysis was performed using IBM SPSS Statistics for Windows, version 26.0 (IBM Corp., Armonk, NY). The kurtosis and skewness values were used to investigate normal distribution. Since the skewness and kurtosis values obtained from the variables were between +3 and -3, normality was ensured, and parametric tests were used (23–25). Chi-square (χ²) test was used for the comparison of categorical data, while independent sample t-test was used for the analysis of parametric variables. The change in YMRS and HAM-D scores from Day 1 to Day 15 of aripiprazole LAI was analyzed with the repeated measures ANOVA test. The effect of initiation regimen on quantitative measurements was analyzed by linear regression test and the effect of initiation regimen on hospitalization history was analyzed by logistic regression test. Data were expressed as mean ± standard deviation (SD) and percent (%) where appropriate. p<0.05 was considered statistically significant. Effect sizes were calculated using Cohen’s d to quantify the magnitude of differences between groups. Cohen’s d values of approximately 0.2 were considered small, 0.5 moderate, and 0.8 or greater large effects. In addition, 95% confidence intervals (95% CI) were reported where applicable to provide an estimate of the precision of effect size and mean difference estimates.

## Results

### Patient demographics and clinical characteristics in TIS vs. OIS groups

TIS (mean ± SD age: 38.8 ± 13.9 years, 56.5% were males) and OIS (mean ± SD age: 40.2 ± 13.1 years, 52.2% were males) groups were homogenous in terms of patient demographics (**Table 1**).

**Table 1:** Patient demographics and clinical characteristics in TIS vs. OIS groups.

Variable	Aripiprazole LAI regimen		P value
	TIS (n=23)	OIS (n=23)	
Age (year), mean ± SD	38.8 ± 13.9	40.2 ± 13.1	0.729^1^
Gender, n(%)			
Male	13(56.5)	12(52.2)	1.000^2^
Female	10(43.5)	11(47.8)
Diagnosis, n(%)			
Psychotic mania	12(52.2)	13(56.5)	1.000^2^
Mixed mania	11(47.8)	10(43.5)
Previous hospitalization, n(%)			
Yes	6(26.1)	14(60.9)	**0.037^2^**
No	17(73.9)	9(39.1)
First attack, n(%)			
Yes	6(26.1)	2(8.7)	0.243^2^
No	17(73.9)	21(91.3)

LAI, Long-acting injectable; OIS, One-injection start; TIS, Two-injection start.

^1^Independent samples t test, ^2^Chi-square test.

Values in bold indicate statistical significance (p<0.05).

Previous hospitalization rate was significantly higher in the OIS group than in the TIS group (60.9% vs. 26.1%, p=0.037) (**Table 1**).

### YMRS and HAM-D scores (Day 1 vs. Day 15) in study groups

There was a significant decrease in YMRS scores from Day 1 to Day 15 of aripiprazole LAI in both TIS (from 24.8 ± 8.7 to 11.0 ± 5.7, p= 0.002) and OIS (from 24.0 ± 8.2 to16.6 ± 6.2, p=0.002) groups, while the change was significantly higher in the TIS group (**Table 2**).

**Table 2:** Study scales in TIS vs. OIS groups.

Scale / Outcome		Aripiprazole LAI regimen		P value^1^	Cohen’s d
		TIS (n=23)	OIS (n=23)		
Scale scores, mean ± SD					
YMRS score	Day 1	24.8 ± 8.7	24.0 ± 8.2	0.755	0.1
	Day 15	11.0 ± 5.7	16.6 ± 6.2	**0.003**	0.9
	p value^2^	**0.002**	**0.002**		
HAM-D score	Day 1	24.3 ± 4.7	23.3 ± 5.2	0.643	0.2
Day 15	13.3 ± 6.5	17.3 ± 5.6	0.127	0.7
p value^2^	**0.025**	**0.025**		
UKU-SERS scores (Day 15)					
Psychic (psychiatric symptom-related) side effects		4.4 ± 1.8	7.5 ± 3.2	**<0.001**	1.2
Neurologic side effects		0.7 ± 1.1	0.8 ± 0.9	0.776	0.1
Autonomic side effects		1.4 ± 1.1	1.9 ± 1.36	0.158	0.4
Other side effects		0.8 ± 0.6	0.6 ± 0.7	0.250	0.3

LAI, Long-acting injectable; OIS, One-injection start; TIS, Two-injection start; YMRS score (range: 0–60): Young Mania Rating Scale; HAM-D-17 score (range: 0–53): Hamilton Depression Scale; UKU-SERS: Udvalg for Kliniske Undersøgelser Side Effect Rating Scale(items are rated from 0 (absent) to 3 (severe); higher subscale scores indicate greater side-effect burden).

^1^Independent Samples t test, ^2^Repeated measure ANOVA test.

Values in bold indicate statistical significance (p<0.05).

There was a significant decrease in HAM-D scores from Day 1 to Day 15 of aripiprazole LAI in both TIS (from 24.3 ± 4.7 to 13.3 ± 6.5, p= 0.025) and OIS (from 23.3 ± 5.2 to 17.3 ± 5.6, p=0.025) groups, while the change was significantly higher in the TIS group (**Table 2**).

### YMRS and HAM-D scores in TIS vs. OIS groups

YMRS scores on Day 15 (11.0 ± 5.7 vs. 16.6 ± 6.2, p=0.003) and UKU-SERS psychic (psychiatric symptom-related) side effects score for psychiatric adverse effects on Day 15 (4.4 ± 1.8 vs. 7.5 ± 3.2, p<0.001) were significantly lower in the TIS group than in the OIS group. Cohen’s d value indicated a large (0.9) and a very large (1.2) effect size for the inter-group difference observed in YMRS Day 15 and UKU-SERS psychic (psychiatric symptom-related) side effects scores, respectively (**Table 2**).

### Linear and logistic regression analyses

Linear regression analysis revealed that OIS regimen was a significant risk factor for increase in YMRS Day 15 scores (B:0.434, p=0.003) and increase in UKU-SERS psychic (psychiatric symptom-related) side effects score (B:0.526, p<0.001) after aripiprazole LAI. Logistic regression analysis revealed that OIS regimen was an independent determinant of previous hospitalization history (OR 4.407, p=0.020), increasing the likelihood of previous hospitalization by 4.407 times compared to TIS regimen.

### Concomitant treatments

Concomitant treatments received by patients in the TIS and OIS groups are summarized in **Table 3**.

**Table 3:** Concomitant treatments in TIS and OIS groups.

Concomitant medication	Aripiprazole LAI regimen	
	TIS (n=23)	OIS (n=23)
Concomitant medications, n(%)		
Atypical antipsychotics	22 (95.7)	17 (73.9)
Mood stabilizers	12 (52.2)	16 (69.6)
Typical antipsychotics	1 (4.3)	3 (13.0)
Benzodiazepines	4 (17.4)	1 (4.3)
SSRI	4 (17.4)	2 (8.7)
SNRI	1 (4.3)	0 (0.0)
Atypical antidepressants	2 (8.7)	2 (8.7)

LAI, Long-acting injectable; OIS, One-injection start; TIS, Two-injection start; SSRI, Selective Serotonin Reuptake Inhibitors; SNRI, Serotonin-Norepinephrine Reuptake Inhibitors

### Safety data

The side effects observed on Day 15 of aripiprazole LAI in TIS and OIS groups are detailed in **Table 4**. Daytime sedation (17.3% in the TIS group and 21.7% in the OIS group) was the most common side effect in both groups. Side effects were tolerable in both groups, and no life-threatening side effects were reported.

**Table 4:** Side effects on Day 15 of aripiprazole LAI injection in TIS and OIS groups.

Adverse event	Aripiprazole LAI regimen	
	TIS (n=23)	OIS (n=23)
Day 15 - side effects, n(%)		
Daytime sedation	4 (17.3)	5 (21.7)
Insomnia	1 (4.3)	2 (8.6)
Forgetfulness	0 (0.0)	1 (4.3)
Increased appetite	2 (8.6)	0 (0.0)
Loss of appetite	0 (0.0)	2 (8.6)
Constipation	2 (8.6)	1 (4.3)
Nausea/vomiting	1 (4.3)	2 (8.6)
Tachycardia	1 (4.3)	2 (8.6)
Epistaxis	0 (0.0)	1 (4.3)
Tension/inner restlessness	1 (4.3)	4 (17.3%)
Difficulty concentrating	1 (4.3)	2 (8.6%)
Tremor	1 (4.3)	3 (13.0)
Paresthesia	1 (4.3)	2 (8.6)
Akathisia	2 (8.6)	2 (8.6)

LAI, Long-acting injectable; OIS, One-injection start; TIS, Two-injection start.

## Discussion

This retrospective, naturalistic, and exploratory study compared the early clinical efficacy and tolerability profiles of the two-injection start (TIS) and one-injection start (OIS) initiation regimens of long-acting injectable (LAI) aripiprazole in patients with bipolar disorder (BD) experiencing manic episodes with psychotic or mixed features. The findings highlight several significant advantages of the TIS regimen, particularly in terms of more rapid symptom control and a better side effect profile.

The reduction in YMRS and HAM-D scores was significantly greater in the TIS group compared to the OIS group during the 15-day early follow-up period. This result can be considered a clinical reflection of the TIS protocol reaching therapeutic plasma levels more rapidly. From a pharmacological perspective, aripiprazole acts as a partial dopamine D2 receptor agonist, 5-HT1A agonist, and 5-HT2A antagonist, thereby modulating both dopaminergic and serotonergic neurotransmission. This mechanism of action contributes to mood stabilization by exerting balanced effects on both manic and depressive symptoms. In the TIS regimen, administration of two 400 mg injections on the same day allows the drug to reach therapeutic plasma levels more rapidly, eliminating the need for 14 days of oral supplementation and potentially enabling faster clinical stabilization. These pharmacokinetic advantages are consistent with the modeling data reported by Martiadis et al. (17) who described comparable but more rapid attainment of steady-state concentrations with the TIS regimen compared with OIS. The faster reduction in symptoms observed in the TIS group aligns with prior research, which suggests that higher initial doses of antipsychotic medication may contribute to earlier symptom improvement. This rapid stabilization can be crucial for patients experiencing acute manic and mixed symptoms, as it reduces the risk of adverse outcomes such as hospitalization or harm to self and others.

Additionally, the side effect profile observed in the TIS group was generally more favorable compared to the OIS group. In terms of tolerability, no statistically significant differences were observed between the TIS and OIS regimens. Nevertheless the UKU-SERS revealed significantly fewer psychiatric adverse effects in the TIS group, which may be attributed to the reduced need for oral supplementation and more consistent plasma levels of aripiprazole. This improved tolerability is consistent with the hypothesis that reducing the number of oral doses required during the initiation phase may reduce treatment burden and facilitate treatment initiation (26). Both initiation strategies were overall well tolerated and no serious or life-threatening adverse events were reported. The logistic regression analysis indicated that patients in the OIS group were more likely to have a history of hospitalization, which might suggest that patients with more severe or treatment-resistant BD were more frequently allocated to the OIS regimen.

This could have impacted the differences in treatment response between the two groups.

The most common adverse events reported in the TIS group were sedation and increased appetite, while in the OIS group, sedation and tension were more prevalent. Importantly, none of these side effects were deemed severe, and they were generally well-managed with standard medical interventions. The absence of significant neurological or autonomic side effects in either group further supports the safety profile of aripiprazole in both OIS and TIS initiation regimens.

Our findings align with the safety profile of aripiprazole TIS regimen reported in previous studies. Cuomo et al. reported that the side effects of TIS regimen were generally mild and manageable (27). Sungur et al. investigated the TIS regimen of aripiprazole in eight severe mania cases with psychotic and mixed features (28). Despite the small sample, TIS regimen was suggested to be well-tolerated and effective, besides its potential to shorten the duration of hospitalization (28).

Several limitations should be acknowledged. First, the retrospective and nonrandomized design limits causal inferences. The relatively small sample size restricts the generalizability of the findings. Future studies with larger, more diverse patient populations are needed to justify the effectiveness and favorable safety profile as well as long-term benefits of TIS regimen in BD patients. Furthermore, the 15-day follow-up period was short and primarily reflects early symptom improvement rather than longterm outcomes. The imbalance in prior hospitalization history between groups may have introduced a potential confounding effect. In addition, concomitant pharmacological treatments were not controlled. Therefore, these findings should be regarded as preliminary and hypothesis-generating. Future prospective, randomized, and adequately powered studies are warranted to minimize selection bias and to more definitively compare the clinical outcomes of different initiation strategies. Nonetheless, our findings provide preliminary evidence that the TIS regimen of aripiprazole LAI may offer an effective and well-tolerated option for patients with BD, particularly in clinical situations where prolonged oral supplementation may be challenging. Another important limitation of this study is the lack of direct measurement of medication adherence, particularly regarding oral aripiprazole supplementation in the OIS regimen. Since adherence was not objectively assessed, differences in clinical outcomes between the OIS and TIS groups may have been partially influenced by unmeasured non-adherence during the initiation phase. Consequently, the findings should be interpreted with caution, and future prospective studies incorporating standardized adherence measures are warranted. In addition, the non-randomized and retrospective nature of the study introduces the possibility of selection bias, as treatment allocation to the OIS or TIS regimen was based on clinical judgment rather than random assignment. This may have resulted in systematic differences between groups that could influence treatment outcomes, such as illness severity or prior treatment history. Although matching was performed to reduce confounding, residual bias cannot be excluded. Therefore, the observed differences between initiation strategies should be interpreted cautiously.

## Conclusion

In conclusion, our findings indicate significant clinical advantages of the TIS regimen of aripiprazole LAI over the traditional OIS regimen in the management of acute manic and mixed episodes in patients with BD. Reduced need for oral supplementation and more consistent plasma levels of aripiprazole seem to make this regimen a preferable option for patients who may experience difficulties with prolonged oral medication use. Both TIS and OIS regimens demonstrated an overall favorable safety profile, with no severe side effects reported. Hence, TIS regimen seems to be an effective and well-tolerated treatment approach, particularly for patients requiring rapid symptom control or in clinical situations where prolonged oral supplementation may be challenging. Future studies with larger samples are needed to justify the effectiveness and safety of TIS regimen in diverse patient populations and to explore long-term benefits of TIS regimen.

## Key points

Aripiprazole LAI’s two-injection start (TIS) regimen shows faster reduction in manic-depressive symptoms compared to the one-injection start (OIS) regimen in bipolar disorder (BD) patients.

By eliminating the need for prolonged oral supplementation, the TIS regimen may facilitate treatment initiation and contribute to earlier clinical recovery.

## Data Availability

The raw data supporting the conclusions of this article will be made available by the authors, without undue reservation.
